# The optic strut: an easily overlooked structure that might cause vision loss

**DOI:** 10.1097/JS9.0000000000004788

**Published:** 2026-01-13

**Authors:** Zirong Chen, Hua Zhang, Zhihai Xie, Junyi Zhang, Sijie Jiang, Kelei Gao

**Affiliations:** a Department of Otolaryngology Head and Neck Surgery, Xiangya Hospital of Central South University, Changsha, People’s Republic of China; b Hunan Province Key Laboratory of Otolaryngology Critical Diseases, Xiangya Hospital of Central South University, Changsha, People’s Republic of China; c National Clinical Research Center for Geriatric Disorders, Xiangya Hospital of Central South University, Changsha, People’s Republic of China; d Anatomy Laboratory of Division of Nose and Cranial Base, Clinical Anatomy Center of Xiangya Hospital, Central South University, Changsha, People’s Republic of China

**Keywords:** anatomy, mucocele, optic strut, vision loss

## Abstract

**Objective:**

To discuss the anatomy of the optic strut (OS) and its implications for the diagnosis and treatment of vision loss caused by OS mucocele.

**Methods:**

We collected and analyzed 5 cases of OS mucocele with vision loss treated from 2013 to 2023, and reviewed literatures on nasal mucoceles and orbital apex anatomy. By comparing reported cases, we summarized the OS anatomical characteristics and their effects on patient symptoms. Concurrently, a comprehensive narrative literature review was conducted using PubMed, Embase, and Web of Science databases to identify studies on optic neuropathy caused by mucoceles in the OS and adjacent structures (anterior clinoid process, sphenoid sinus, ethmoid sinus). Relevant data were extracted for descriptive statistical analysis.

**Results:**

All patients were followed up for 6 months postoperatively. One patient showed no significant vision improvement, while the other four had vision recovery: 0.5→1.2, 0.6→1.0, 0.7→1.0, and 1.0→1.2 (typical case). For the narrative literature review, 15 studies encompassing 48 cases were included, with 19 cases analyzed in detail. All 19 cases presented with vision loss (100%), accompanied by headache (68.4%) and afferent pupillary defect (APD, 47.4%) in partial cases. Lesions were confined to the anterior clinoid process in 4 cases and involved the sphenoid sinus in the rest, showing expansile features on CT and high T2 signal on MRI. Endoscopic endonasal surgery was adopted in 63.2% of cases; timely intervention achieved complete visual recovery in 52.6% of patients, whereas delayed treatment frequently led to optic atrophy.

**Conclusions:**

The OS is a bony structure separating the superior orbital fissure, optic canal, and internal carotid artery. OS mucocele–induced pressure elevation and inflammation can damage the optic nerve and cause vision loss. Prompt surgical resection and optic nerve decompression are critical for preserving visual function in patients with space-occupying lesions around the optic canal.

## Introduction

The optic strut (OS), defined as the posterior root of the sphenoid’s lesser wing, serves as a key bony pillar. It constitutes the inferolateral wall of the optic canal and thereby separates the optic nerve from the superior orbital fissure (SOF) and the internal carotid artery (ICA)^[1]^. Pneumatization of the OS can occur secondary to a well-aerated sphenoid sinus or sphenoethmoidal cells. Although rare, localized mucoceles may develop within the pneumatized OS as a result of obstructed drainage. These benign, expansile OS mucoceles can subsequently cause compression and erosion of the neighboring critical neurovascular tissues^[2]^.

In clinical practice, patients with an OS mucocele typically present with visual impairment or headache, often in the absence of nasal symptoms. This clinical picture commonly leads to initial evaluation by ophthalmologists. However, due to its rarity and the fact that the OS remains an underemphasized structure in either ophthalmology or otorhinolaryngology literature, the condition is prone to misdiagnosis as optic neuritis or other common ocular pathologies, resulting in critical delays in appropriate management^[2–4]^.

Given this diagnostic and therapeutic challenge, this study, based on a review of five surgically managed cases and the relevant literature, aims to delineate the clinical anatomy of the OS and its role in compressive optic neuropathy. We also discuss the rationale for prompt surgical intervention, which emphasizes lesion resection and optic nerve decompression to maximize visual recovery. This study was conducted in compliance with the Transparent and Integrative Tool for AI in Neuroscience (TITAN) Guidelines 2025, which govern the declaration and responsible use of artificial intelligence tools in research^[5]^.

## Methods

### Case series selection and data collection

We retrospectively collected and analyzed medical records of patients with OS mucocele treated in the Department of Otorhinolaryngology of our hospital from January 2013 to August 2023. A total of five patients with complete data were included, comprising two males and three females, with an age range of 30–50 years.

All five patients presented with decreased vision, and two had concurrent headaches. Disease duration ranged from 2 weeks to 8 months. Initially, all patients were evaluated in ophthalmology departments, where they were diagnosed as either presbyopia or optic neuritis, treated with antibiotics and hormones, but showed little improvement in vision. After further imaging, four patients were suspected of having space-occupying lesions around the optic canal and referred to our department, while one patient was treated for optic neuritis for 6 months before being referred to our department.

All patients underwent endoscopic lesion resection and decompression of the affected optic nerve. The surgical procedure involved routine ethmoidectomy and sphenoidotomy to fully expose the medial orbital wall. The lesion was identified and removed until the pneumatized OS was exposed, following which the bony walls of the optic canal were removed to decompress the optic nerve. Gelatin foam soaked with methylprednisolone was placed over the nerve postdecompression. Patients received postoperative systemic methylprednisolone, antibiotics, and neurotrophic drugs, alongside topical agents to promote mucosal healing.

### Literature search and selection criteria

A comprehensive narrative literature review was conducted using PubMed/NCBI to identify studies on mucoceles or mucopyoceles causing optic neuropathy or orbital apex syndrome. Given the rarity of lesions specifically originating from the OS, the search was broadened to include adjacent anatomic regions such as the anterior clinoid process, sphenoid sinus, and ethmoid sinus. Search terms included combinations of relevant anatomical and clinical keywords (e.g., “mucocele,” “visual loss,” “optic neuropathy”). Case reports, case series, and small retrospective studies documenting symptomatic patients with visual impairment or ophthalmoplegia were included.

### Data extraction and analysis

Data on demographics, clinical presentation, imaging features, treatment modalities, and visual outcomes were systematically extracted from the eligible studies. Descriptive statistics were used to summarize the clinical characteristics, management strategies, and prognostic factors associated with these cases.

This study was conducted in accordance with the ethical principles of the Declaration of Helsinki and was approved by the local independent ethics committee (NO. 2 023 030 623).

## Results

### Presentation and outcomes of the present case series

All five patients were followed up for 6 months postoperatively. With the exception of one patient, who had an 8-month disease course including 6 months of conservative treatment prior to referral and showed no significant vision improvement, the other four patients all experienced vision recovery: 0.5→1.2, 0.6→1.0, 0.7→1.0, and 1.0→1.2. A detailed summarization of these five cases is presented below (Table 1). The nasal cavity and paranasal sinuses showed good epithelization, with no vesicles or polyps observed.

**HIGHLIGHTS**

- **Anatomical Insight**: The optic strut (OS), a critical bony structure separating the superior orbital fissure, optic canal, and internal carotid artery, forms the inferior wall of the optic canal and is pivotal in understanding orbital apex pathologies.
- **Clinical Relevance**: Pneumatization of the OS and anterior clinoid process can lead to mucoceles, causing vision loss via direct compression and inflammatory damage to the optic nerve, often misdiagnosed as ophthalmic conditions like optic neuritis.
- **Surgical Efficacy**: Endoscopic resection of OS mucoceles combined with optic nerve decompression achieved significant visual recovery in four out of five patients (follow-up ≥6 months), with improvements from 0.5→1.2, 0.6→1.0, 0.7→1.0, and 1.0→1.2.
- **Diagnostic and Therapeutic Guidelines**: Unexplained vision loss requires CT/MRI to identify perioptic space-occupying lesions. Early surgical intervention (complete lesion removal and optic canal decompression) is advocated to preserve visual function, as optic nerve regeneration is limited.
- **Anatomical Correlation**: Symptom presentation of anterior clinoid process mucoceles is linked to pneumatization origin – lesions arising from the OS tend to cause earlier vision loss compared to those from the upper root.

**Table 1:** 

Number	Case 1	Case 2	Case 3	Case 4	Case 5 (typical case)
Age (years old)	31	50	45	49	39
Gender	F	M	M	F	F
Chief complaint	Headache, decreased visual acuity	Recurrent decreased visual acuity	Decreased visual acuity	Decreased visual acuity	Decreased visual acuity
Associated symptoms	Visual field defect	Intermittent headache,	Visual field defect, color vision loss	None	None
Disease duration	14 days	8 months	1 month	2 months	8 months
Preoperative visual acuity	Left eye: 0.5, right eye: 1.0	Left eye: 1.2 (corrected), right eye: 0.6 (corrected)	Left eye: 0.6, right eye: 1.0	Left eye: 1.2 (corrected), right eye: 0.7 (corrected)	Left eye: 1.2 (corrected), right eye: 1.0 (corrected)
Preoperative visual field	Left eye: partial inferotemporal visual field defect	Normal	Normal	Normal	Right eye: partial upper visual field defect
Postoperative visual acuity	Left eye: 1.2, right eye: 1.0	Left eye: 1.2 (corrected), right eye: 0.6 (corrected)	Left eye: 1.0, right eye: 1.0	Left eye: 1.2 (corrected), right eye: 1.0(corrected)	Left eye: 1.2 (corrected), right eye: 1.2 (corrected)
Preoperative VEP	Left eye: visual conduction dysfunction	Right eye: visual conduction dysfunction	Right eye: visual conduction dysfunction	Right eye: visual conduction dysfunction	Right eye: visual conduction dysfunction
Postoperative VEP	Left eye: normal visual conduction function	Unchanged visual conduction dysfunction	Right eye: normal visual conduction function	Right eye: normal visual conduction function	Right eye: normal visual conduction function
Time for improvement	1 month after surgery	No improvement in visual acuity	1 week after surgery	2 weeks after surgery	10 days after surgery
Postoperative complications	None	None	None	None	None

### Typical case illustration

**Figure 1.**
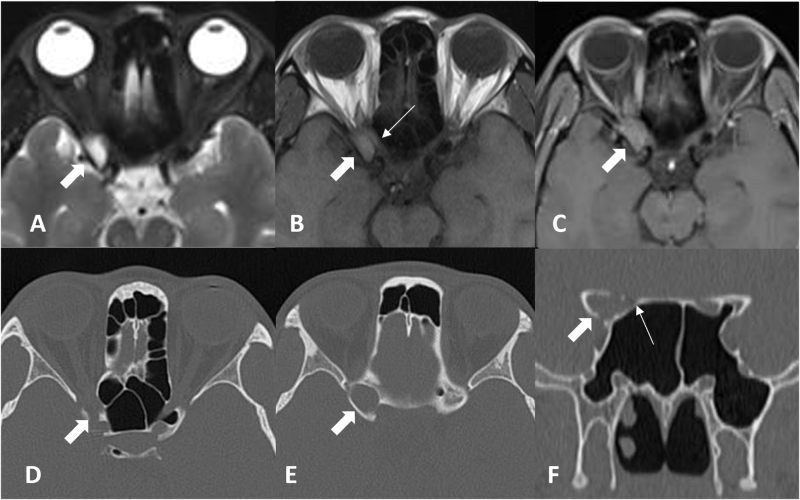
Preoperative MRI and CT results: (A) Axial T2, (B) Axial T1, and (C) Axial T1 with contrast: showing an expansile lesion at the orbital apex (thick arrows) with slightly hyperintense T1 signal, hyperintense T2 signal, and peripheral marginal enhancement on contrast-enhanced T1; thin arrow in (B) indicates the compressed right optic nerve. (D) Axial CT, (E) Axial CT, (F) Coronal CT: showing the expansile mucocele as soft tissue density (thick arrows) with bony thinning of the OS and ACP; thin arrow in (F) indicates the compressed right optic canal.

**Figure 2.**
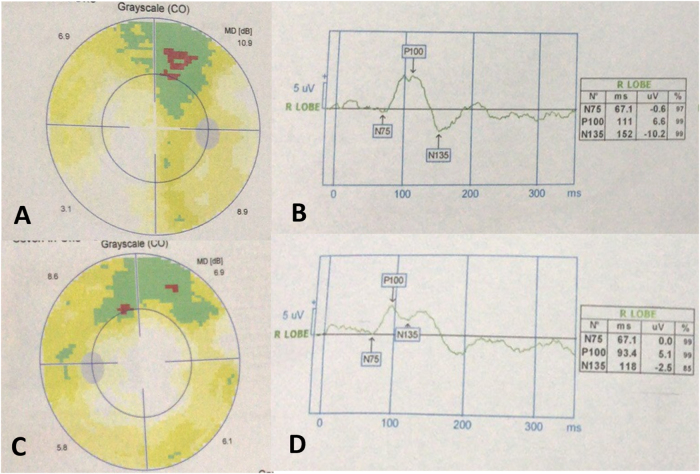
Preoperative eye examinations: (A, B). Right eye: (A) Visual field test showing slightly reduced upper field; (B) VEP showing P100 signal latency. (C, D) Left eye: showing intact visual function.

**Figure 3.**
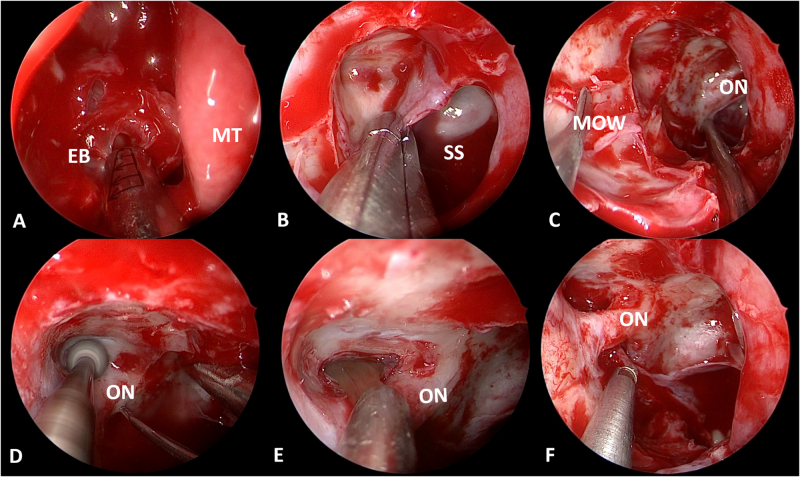
Surgical procedures: (A) Ethmoidectomy; (B) Sphenoidotomy; (C) Resection of part of the medial orbital wall to expose the orbital apex. (D) Grinding of the optic canal’s bony walls; (E) Removal of mucocele contents; (F) Resection of residual mucocele walls. MT, middle turbinate; EB, ethmoid bulla; SS, sphenoid sinus; MOW, medial orbital wall; ON, optic nerve.

A 39-year-old female presented with an 8-month history of right-eye vision loss, without headache, diplopia, ptosis, or visual field defects. She initially visited an ophthalmology department, where she was diagnosed with presbyopia. As her vision loss progressed rapidly, further imaging revealed a space-occupying mass around the orbital apex (Fig. 1), and she was referred to our department. Her visual acuity was 1.0 in the right eye. Additional ophthalmic examinations showed impaired right-eye function: slightly reduced upper visual field and mild P100 signal latency in visual evoked potential (VEP) testing (Fig. 2). Surgery was performed as described above (Fig. 3). Postoperatively, her vision recovered to 1.2.

### Findings from the comprehensive narrative literature review

This study collected data from 15 studies reporting on 48 unique cases of optic neuropathy caused by mucoceles of the anterior clinoid process (ACP) or sphenoid sinus^[2,3,6–29]^. A detailed analysis of 19 cases is presented below (Table 2).

**Table 2:** 

Source	Age, sex	Underlying diseases & risk factors	Clinical presentation & disease course	Diagnostic methods (Imaging findings)	Treatment details	Specific visual acuity change (preoperative → postoperative)	Lesion location (anterior clinoid process involvement)	Other outcomes
Abozed *et al* (2017)^[3]^	66, M	Not specified	Acute left eye visual loss + headache	CT/MRI: Left ACP mucocele compressing optic nerve	Emergency surgical resection and decompression	Light perception → 20/25 (complete recovery)	Yes (ACP)	None
Medina-Valencia *et al*. (2022)^[22]^	62, F	No significant history	Progressive monocular visual loss in the right eye over 2 years	CT: Pneumatized right ACP + cyst compressing the optic canal	Endoscopic transethmoidal resection	Not specified quantitatively → complete recovery	Yes (ACP)	None
O’Donnell *et al*. (2022)^[30]^	37, M	None	Fever, left eye visual decline, cranial nerve palsy	CT: Left ACP pyocele	Craniotomy after failed antibiotics	Visual decline → complete symptom resolution (acuity not quantified)	Yes (ACP)	Other symptoms resolved.
Nundkumar *et al* (2012)^[16]^	32, M	Not specified	Acute, painless left eye visual loss	MRI: Left ACP cyst compressing the optic nerve	Endoscopic endonasal decompression	No light perception (NLP) → 20/20 (Complete recovery)	Yes (ACP)	None
Johnson *et al* (1986)^[14]^	56, M	Suspected diabetes	Diplopia, fluctuating visual loss	CT: ACP expansion/erosion	Surgical resection	Not specified → partial recovery	Yes (ACP)	None
Kiko *et al*. (2020)^[21]^	63, F	Not specified	Diplopia + left ptosis, acute visual loss after 6 months (1-week duration)	MRI: Left ACP cyst growth	Craniotomy for cyst resection	Visual loss → significant recovery (not quantified)	Yes (ACP)	None
Zhang *et al*. (2020)^[20]^	64, F	Not specified	Acute, painless monocular visual loss (left eye)	CT/MRI: Left ACP mucocele compressing optic nerve	Surgical decompression (details unspecified)	Preoperative NLP → No recovery at 6 months	Yes (ACP)	None
Park *et al* (2023)^[24]^	17, M	Pyogenic infection (MRSA bacteremia), hypopituitarism	Orbital apex syndrome, left eye NLP, fever, headache	MRI: Sphenoid sinus mucocele compressing the orbital apex	Surgery + antibiotics/steroids	NLP → No recovery	No (Sphenoid Sinus)	Persistent endocrine abnormalities.
Nadeem *et al*. (2021)^[25]^	63, F	Hypertension	Sudden left eye visual loss with periorbital pain (1-month history)	Imaging: Left sphenoid sinus mucocele compressing the optic nerve	Endoscopic sinus surgery (delayed diagnosis)	Visual loss → No recovery (optic atrophy)	No (Sphenoid Sinus)	Irreversible damage
Ma Yi *et al* (2010)^[26]^	30 cases (mean 41)	Some with chronic sinusitis	Headache (80%), visual disturbance (63.3%)	CT/MRI: Sphenoid sinus cyst	Endoscopic sinus surgery	e.g., Blindness → improvement in 7 cases; No improvement in 2 cases (duration >1 month)	No (Sphenoid Sinus)	Majority improved
Liu Feng *et al* (2025)^[27]^	4 cases (15–58)	1 case with chronic sinusitis	Visual decline + headache (misdiagnosed)	CT/MRI: Sphenoid sinus occupying lesion	Emergency endoscopic drainage	Case1: light perception → 0.1; Case2: –blindness/counting fingers → 0.2	No (Sphenoid Sinus)	Variable improvement
Sundar *et al* (2004)^[28]^	22, M	Not specified	Acute, painless monocular visual loss	MRI: Sphenoid sinus mucocele	Endoscopic surgery + steroids	NLP → 6/6 within 48 hours (complete recovery)	No (Sphenoid Sinus)	None
Levy *et al* (2005)^[29]^	Not specified	Not specified	Right eye long-term blindness, followed by acute left eye visual loss	Imaging: Sphenoid sinus mucocele	Emergency endoscopic decompression	Left eye: acute loss → partial recovery	No (Sphenoid Sinus)	None
Fukuda *et al*. (2010)^[23]^	Not specified	Not specified	Chronic left eye visual decline	MRI: Onodi cell mucocele	Pterional epidural approach resection + decompression	Visual decline → significant improvement to 20/16	No (Onodi cell)	None
Jiang *et al* (2011)^[31]^	23 cases	Some with sinusitis	Visual decline, diplopia, headache	CT/MRI: Sinonasal lesions	Endoscopic exploration/decompression	Significant improvement with early surgery (values not unified)	No (Paranasal Sinuses)	Overall improvement

### Clinical presentation and imaging features

Visual loss (VL) was the universal presenting symptom (100%). Headache or pain was a common accompanying feature, present in 68.4% (13/19) of cases, and specific findings such as an afferent pupillary defect (APD) were noted in 47.4% (9/19)^[3,6–14,16–23]^. Neuroimaging was critical for diagnosis. Computed tomography (CT) consistently revealed a pneumatized ACP with an associated expansile, erosive, nonenhancing mass^[3,6–14,16–19,22]^. Magnetic resonance imaging (MRI) typically showed a cystic lesion, hyperintense on T2-weighted images with variable T1 signal and no significant contrast enhancement, aiding differentiation from neoplastic lesions^[3,9,13,15,18,19]^.

### Treatment and prognosis

Surgical decompression was the primary treatment. The endoscopic endonasal approach was the most frequently employed technique, used in 63.2% (12/19) of cases^[3,6,9,11,12,14–18]^. Alternative approaches included pterional^[9,13,19]^ and supraorbital craniotomies^[7,8]^. Visual prognosis was strongly correlated with intervention timeliness. A “full recovery” of vision was documented in 52.6% (10/19) of cases, all of which involved prompt surgical intervention^[6,9,11,12,14,15,17,18]^. In contrast, delayed treatment or presentation with severe, long-standing vision loss often resulted in suboptimal outcomes, such as only “slight improvement” or “no recovery,” with subsequent optic nerve atrophy^[8,13,19]^.

## Discussion

### Anatomy and adjacency of the OS

**Figure 4.**
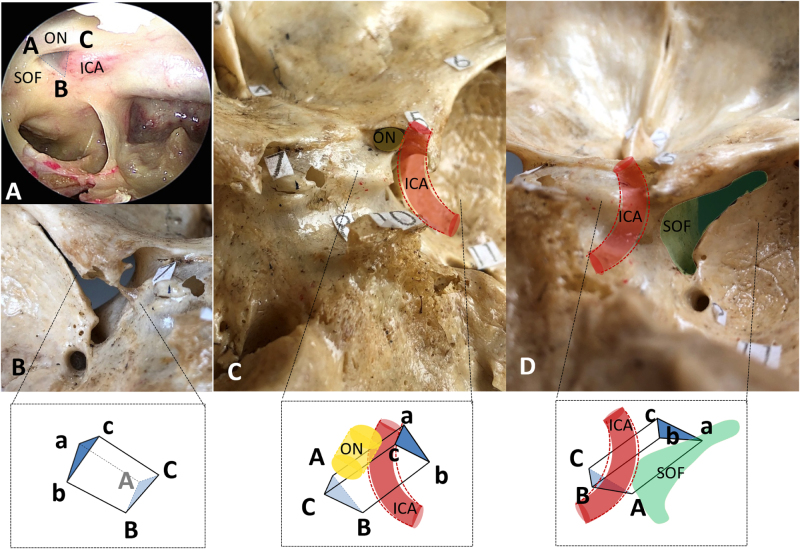
Anatomy of the optic strut: (A) Endoscopic view showing triangle ABC (base of the pneumatized prism) of the right OS; (B) Intracranial view of the left OS, with top triangle abc and posterior surface bBCc facing the observer; (C) Intracranial view of the right OS, with upper surface aACc and posterior surface bBCc facing the observer; (D) Intracranial view of the right OS, with posterior surface bBCc and lower surface aABb facing the observer. ON, optic nerve; ICA, internal carotid artery; SOF, superior orbital fissure.

The ACP is connected to the sphenoid body via upper and lower roots. The flat upper root forms the optic canal’s superior wall and extends inward to the planum sphenoidale. The lower root is the OS, which forms the optic canal’s inferior wall and separates it from the SOF. The OS can be roughly described as a triangular prism with three sides and two ends (attaching to the sphenoid body and ACP)^[32]^. When pneumatized, this “prism” hollows into the opticocarotid recess (OCR), visible endoscopically (Fig. 4).

The OS’s lower surface forms the superior part of the SOF’s inner wall; its upper and lower edges divide the SOF into three segments: lateral, middle, and lower^[1]^. The lateral segment contains the lacrimal nerve, frontal nerve, trochlear nerve, and superior ophthalmic vein (adjacent to the lateral rectus muscle). The middle segment corresponds to the oculomotor foramen – a soft tissue channel formed by fibers/tendons of the superior/lateral rectus muscles and their interligaments – through which the oculomotor nerve, nasociliary nerve, and abducens nerve pass^[33]^. The lower segment lies between the oculomotor foramen and Müller’s muscle, allowing intraorbital fat to extend posteriorly into the infraorbital fissure (IOF).

The OS’s posterior surface abuts the clinoid segment of the ICA. Near the ACP, the upper edge of this surface aligns with the ICA’s distal ring (a fibrous ring formed by dura mater and clinoid periosteum), while the lower edge aligns with the ICA’s proximal ring [a frequently incomplete fibrous ring formed by the ICA-oculomotor membrane (COM) and adjacent periosteum]. The ICA’s clinoid segment is bounded by these two rings and may be surrounded by a clinoid venous plexus communicating with the cavernous sinus^[34]^. Near the upper edge, the ICA gives rise to the ophthalmic artery, which runs through the optic canal between the OS and optic nerve^[1,30]^.

The OS’s upper surface forms the optic canal’s floor, upon which the optic nerve and ophthalmic artery (lying beneath the nerve) rest^[30]^. The optic canal runs between the sphenoid body and ACP: its roof comprises the ACP’s upper root, the sphenoid lesser wing, and the sickle ligament^[35]^; its lateral wall comprises the OS and part of the ACP; its medial wall is the lateral wall of either the ethmoid or sphenoid sinus (depending on their competitive pneumatization). Due to incomplete overlap between the canal’s floor and roof, its length remains controversial^[1,30]^. Unlike the contents of other OS-adjacent spaces, the optic canal’s contents are enclosed in a complete bony channel, making them highly sensitive to surrounding pressure changes.

### Significance of OS mucocele

Like other pneumatized nasal cells, the pneumatized OS (and even ACP) can develop mucoceles. Etiologies include poor drainage from mucosal inflammation, cyst formation in nasal epithelial glands, nasal polyps^[14]^, or ectopic epithelial entrapment during bone development^[36]^. Sphenoid sinus or sphenoethmoidal cell mucoceles are rare (1%–2% of all sinus mucoceles)^[37]^, and pneumatized OS/ACP mucoceles are even less common. Since the first report in 1986^[14]^, fewer than 20 cases have been described in just over 10 publications addressing this condition^[31]^. Reported symptoms include headache, vision loss, eye movement disorders^[12]^, and supraorbital/frontal sensory abnormalities^[12]^. Potential mechanisms of vision loss are: (1) Direct mucocele compression causing optic nerve ischemia and edema; (2) Local inflammatory factors from the mucocele stimulating the optic nerve via absorbed bone and periosteum^[16]^.

Pressure-induced nerve ischemia and subsequent edema form a vicious cycle, but not all patients develop vision loss. Tchoyoson *et al* reported a 61-year-old male with sudden diplopia and forehead numbness^[38]^; O’Donnell *et al* reported a 37-year-old male with headache, diplopia, and ptosis^[39]^; Kwon *et al* reported a 62-year-old male with downward diplopia^[40]^ – none had vision loss. CT scans of these cases showed mucocele involvement only in the ACP’s upper root (optic canal roof), not the OS (optic canal floor).

**Figure 5.**
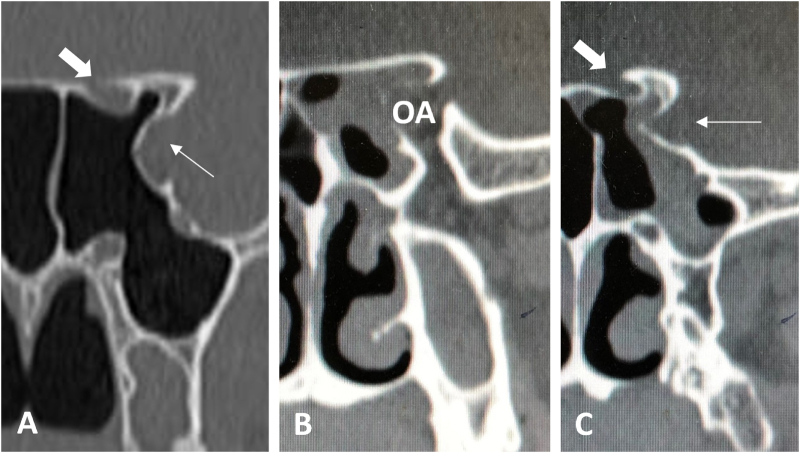
Potential pneumatization pathways of the anterior clinoid process: (A) Axial CT of the left orbital apex showing pneumatized ACP and OS; (B). Pneumatized and involved upper root of the ACP (showing adjacent structure involvement); (C). Pneumatized and involved OS (showing structure involvement). Thick arrows indicate optic canals; thin arrows indicate superior orbital fissures (A, C); OA, orbital apex.

Mikami *et al* noted that 74.5% of ACP pneumatization originates from the OS, 14.5% from the upper root, and 10.9% from both^[41]^. Combined with OS anatomy, we hypothesize that ACP mucocele symptoms correlate with its origin: when mucocele arises from the pneumatized upper root/ACP, early pressure can be relieved into the cranial cavity, often without optic nerve compression or vision loss. As the disease progresses, the optic canal and SOF’s lateral segment become involved, leading to vision loss, supraorbital sensory abnormalities, and occasionally diplopia^[40]^. When a mucocele arises from the OS, the mucocele directly compresses the optic nerve against the ACP’s upper root, causing early vision-related symptoms; subsequent involvement of the SOF’s middle (eye movement disorders) or lateral (sensory abnormalities) segment determines additional symptoms (Fig. 5).

### Diagnosis and treatment strategy of OS-related ACP mucocele

Patients typically present with ophthalmic symptoms (no nasal symptoms like congestion or purulent discharge) and first seek ophthalmic care. Due to the condition’s rarity and complex regional anatomy, inexperienced ophthalmologists may struggle to differentiate it from optic neuritis, macular degeneration, or glaucoma – delaying treatment. Thus, for unexplained vision loss, the possibility of perioptic space-occupying lesions should be considered, and CT/MRI should be performed^[42]^. On CT, the lesion appears as a soft tissue mass involving the OS, ACP, and adjacent structures, with bony compression/absorption around the ACP and OS^[40]^. MRI reflects the mucocele's content nature: T1 signal increases and T2 signal decreases with water absorption^[38]^, and peripheral enhancement may be seen along the mucocele wall.

Early literature suggested conservative treatment (antiinflammation, antiinfection, neurotrophy) first for timely diagnosed OS mucocele, with surgery reserved for refractory cases^[10,39]^. However, the optic nerve has extremely poor regenerative capacity, and symptom alleviation may take weeks to months – rendering a “wait-and-see” approach impractical^[43]^. Thus, we advocate for immediate surgery once imaging confirms diagnosis: lesion resection and nerve decompression eliminate perioptic inflammatory stimulation, relieve pressure, and improve local nerve blood circulation.

Key surgical considerations based on OS anatomy:

- Total ethmoidectomy and sphenoidotomy create surgical space and expose anatomical landmarks [e.g., bony ridge holding the posterior ethmoidal artery, orbital apex, OCR (pneumatized OS)].
- Unlike ethmoidal cell mucoceles (curable by partial cyst wall resection), ACP mucocele requires complete removal of contents and all cyst walls occupying the OS/ACP to fully expose the optic canal; at least half of the canal’s bony walls should be ground away for nerve decompression^[44]^.

(c) Incising the optic nerve sheath is not recommended, as it may impair nerve blood supply^[45]^.

(d) The OS’s posterior surface abuts the ICA and its ophthalmic branch; bony absorption of this surface (in some cases) increases ICA vulnerability. Preoperative CT must be carefully evaluated to assess the risk of ICA exposure, and sharp instruments should be avoided near the OCR to prevent vascular injury.

Perioperative conservative measures (neurotropin, glucocorticoids for antiinflammation/edema) are essential; hyperbaric oxygen therapy and dehydrants may be beneficial in some cases^[31]^.

Prognosis varies across the literature: some cases showed no full recovery despite complex treatment^[31]^, while others achieved gradual full recovery at 1 month^[46]^, 4 months^[40]^, or 6 months^[3]^. In our series, four patients had slow but full vision recovery after a 6-month follow-up. Prognostic differences may relate to disease duration, prior treatment, lesion location, and surgical technique. However, both literature and our experience confirm that even without immediate postoperative improvement in vision/eye movement, optic nerve function rehabilitation and conservative treatment remain necessary.

## Conclusions

The OS is a critical bony structure that delineates the SOF, optic canal, and ICA; it forms the optic canal’s floor and part of its lateral wall. Mucoceles may develop in the pneumatized OS and adjacent ACP, and increased pressure plus inflammatory factors can impair the optic nerve, causing vision loss. For patients with unexplained vision loss, further imaging is recommended after excluding other ophthalmic diseases. For space-occupying lesions around the optic canal, prompt surgical intervention, including lesion resection and optic nerve decompression, is critical to preserve visual function.

## Data Availability

Data sharing is not applicable to this article.

## Statement

We confirm that this article complies with the TITAN Guidelines 2025, which govern the declaration and use of artificial intelligence (AI).
